# The first study on the prevalence of gastrointestinal parasites in owned and sheltered cats in Yangon, Myanmar

**DOI:** 10.14202/vetworld.2023.414-420

**Published:** 2023-02-28

**Authors:** Babi Kyi Soe, Khin Su Hlaing, Toe Win Naing, Zin Hnin Thaw, and Win Myint

**Affiliations:** 1 Department of Livestock Breeding and Veterinary, Yangon 11021, Myanmar; 2 Livestock Improvement Section, De Heus Myanmar, Yangon 11021, Myanmar; 3 Pathology Unit, Department of Livestock Breeding and Veterinary, Yangon 11021, Myanmar

**Keywords:** cat, gastrointestinal parasites, Myanmar, prevalence

## Abstract

**Background and Aim:**

People who used to rear companion animals are healthier than others who do not. Gastrointestinal (GI) helminths are common in cats and serve as reservoirs for zoonotic diseases. However, the prevalence of GI parasites in cats in Myanmar has never been reported. This study aimed to estimate the prevalence of GI parasites in cats in Myanmar and to identify the potential risk factors associated with GI parasites.

**Materials and Methods:**

A total of 230 fecal samples were collected from seven veterinary clinics and two shelters within the Yangon region from January to May 2022. Sampled cats were classified according to age, gender, and the deworming and rearing practices. Fecal samples were analyzed by fecal wet mount, ethyl acetate centrifugal sedimentation, and zinc sulfate centrifugal flotation techniques. Descriptive data were described, and Pearson’s χ^2^ test was used to identify associated risk factors, such as age, gender, and the deworming and rearing practices.

**Results:**

The overall prevalence of GI parasites was 79.56%, and 57.82% of cats were infected with a diagnostic stage of more than one parasite species. Seven GI parasites were detected, including *Ancylostoma* spp. (55.65%), *Toxocara* spp. (46.08%), *Trichuris* spp. (20.86%), *Platynosomum* spp. (11.73%), *Dipylidium caninum* (7.39%), *Taenia* spp. (4.34%), and *Cystoisospora* spp. (32.17%). Based on statistical analysis, the deworming and rearing practices were significantly associated (p < 0.05) with GI parasitic infections.

**Conclusion:**

This study is the first to reveal the prevalence of GI parasites that could assist the need for effective control measures for zoonotic hookworm and roundworm infections in cats. Even with simple microscopic examination, the remarkably high prevalence of GI parasitic infections warrants regular deworming practice. Further molecular studies should also be performed to understand their genetic diversity.

## Introduction

People who used to rear companion animals are healthier than others who do not [1]. Gastrointestinal (GI) helminths are common in companion animals, particularly cats [2]. More than 3 billion people are affected by parasitic infections yearly worldwide [3], and companion animals serve as reservoirs to complete the parasite life cycle. Domestic cats are considered as intimate companions of humans. Likewise, they act as reservoirs for zoonotic diseases through contamination with their infected feces [4]. Although companion animals have shown health benefits for humans, they play a critical role in parasite transmission. However, few data on parasitism are available for cats compared to dogs [5]. For example, *Ancylostoma* spp. and *Toxocara* spp. of domestic cats have public health significance [6]. Other parasites have feline zoonotic importance, such as *Giardia* spp., *Cryptosporidium* spp., *Echinococcus* spp., *Sarcocystis* spp., *Blastocystis* spp., and *Strongyloides* spp. [7, 8]. Previously, the development of the infective stage of *Ancylostoma* spp. and *Toxocara* spp. has been reported in favorable climatic conditions of the neighboring country, Thailand [9]. Parasitized cats may show various symptoms according to parasite species and intensity. Cats may infect subclinically and sometimes show the symptoms of dull coat, vomiting, diarrhea, and weight loss, eventually leading to death in chronic infections [10]. In addition, parasitized cats have become more susceptible to other bacterial and viral infections [11]. An epidemiological survey of GI parasites in cats has been performed worldwide to promote control measures. Based on the literature, the prevalence of GI parasites in domestic cats from different countries (i.e., Indonesia, Thailand, Malaysia, India, Korea, Japan, and China) using coprological examinations are described in Table-1 [12–18].

**Table-1:** Prevalence of gastrointestinal parasites in domestic cats from different countries.

Countries	Prevalence % (n)	Diagnosis method	Reference
China	41.39 (149/360)	Sugar flotation	[12]
Malaysia	89.3 (25/28)	Direct smear and ethyl acetate concentration	[13]
India	100 (100/100)	Sedimentation, flotation, and Mc Master technique	[14]
Indonesia	68.33 (88/120)	Direct smear, sedimentation, and flotation	[15]
Korea	39.8 (162/407)	Flotation with saturated sodium chloride	[16]
Thailand	44.3 (133/300)	Formalin ether concentration	[17]
Japan	20.8 (69/342)	Formalin ethyl acetate sedimentation	[18]

Regarding diagnosis techniques, fecal wet mount, centrifugal sedimentation, and centrifugal flotation are the most common [19]. Briefly, fecal wet mount is used as a rapid screening test, centrifugal sedimentation is used to recover operculated- trematode and nematode eggs, and centrifugal flotation is used for nonoperculated nematode egg recovery [20]. To detect GI parasites from small fecal masses, fecal wet mount was performed first in this study, followed by centrifugal sedimentation and centrifugal flotation. Although there is a close association between cats and humans, little attention has been received on the zoonotic parasites of domestic cats in Myanmar. Hence, a relatively high prevalence of intestinal worm infections has been reported in those who live in riverside regions in Myanmar [21], and there is a lack of data on GI parasitic infections in cat reservoirs.

To date, information on the prevalence of GI parasites in cats has never been reported in Myanmar. The widespread usage of anti-anthelmintic drugs within the downtown culture seemed to reduce parasitic infections in Myanmar. Based on the previous reports of GI parasites in cats from neighboring countries, it is challenging to realize the upcoming relatively high prevalence of GI parasites [10, 12].

Therefore, this study aimed to determine the prevalence of GI parasites in cats, compare the prevalence between the two fecal examination techniques, and identify the potential risk factors associated with GI parasites. The results may serve as a roadmap for feline GI parasitic infections in Myanmar. Considering this, effective control measures for zoonotic diseases can be further provided.

## Materials and Methods

### Ethical approval

Fecal sample collection procedure was performed as per the guidance of Myanmar Veterinary Association in accordance with animal welfare principles together with signed consent form for owned cats. All procedures were approved by Research Development Committee of Livestock Breeding and Veterinary Department with No. 12/2022-LBVD, Myanmar.

### Study period and location

**Figure-1:**
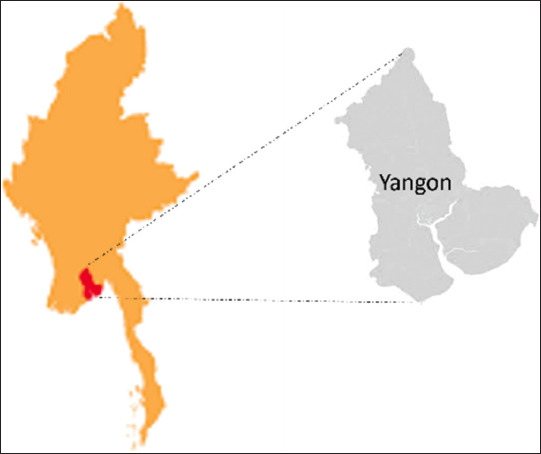
The map of Myanmar (left) and the location map of study area (Yangon region). (Raw map was sourced from https://en.wikipedia.org/wiki/Yangon_Region).

The study was conducted from January to May 2022 in Yangon, the largest city in Myanmar (Figure-1). Yangon region occupies 1875.42 km^2^ and the region is located at 16.8409°N and 96.1735°E of about 23 m above sea level. The average temperature was 30.5°C with 2375 mm of annual rainfall. Fecal samples were collected from a total of 7 veterinary clinics according to a dense population of domestic cats in these clinic sites. Two shelters were involved in this study. Fecal microscopic examination was performed at Parasitology Laboratory, Livestock Breeding and Veterinary Department, Insein, Yangon.

### Sampling and coprological examination

Using the convenience sampling method, 230 fecal samples were collected from owned cats who visited selected veterinary clinics and two shelters. Profiles of the sampled cats, including age, gender, and the deworming and rearing practices, were recorded. In detail, different age groups (<1, 1–3, and >3 years), history of the deworming practice, and outdoor assessments were obtained. Fecal samples were collected directly from the rectum using disposable gloves and placed inside properly labeled ziplocked bags. The ice box (4°C) containing collected fecal samples was carried to the Parasitology Laboratory, Insein, Yangon, for conventional fecal microscopic examination. Before the fecal examination, the fecal samples were refrigerated at 4°C. As described, fecal wet mount, ethyl acetate centrifugal sedimentation, and centrifugal flotation were used to demonstrate GI parasites, including nematodes, cestodes, and trematodes [19, 22]. According to the basic morphological characteristics, the detection of GI parasites was performed. The cats were considered positive when at least one parasite egg was observed by any of the conventional microscopic techniques.

### Statistical analysis

The prevalence rate of GI parasitism was analyzed by descriptive statistics. Pearson’s χ^2^ test was used to test the significance between prevalence and categorical variables, such as age, gender, and the deworming and rearing practices. Calculations were made by SPSS version 17 (IBM Corp., NY, USA). The significance level was set at 0.05 with a 95% confidence interval. p < 0.05 was considered as significant.

## Results

This study collected 230 feline fecal samples from owned cats from seven veterinary clinics and two shelters within the Yangon region, Myanmar. The profile of sampled cats was categorized into four main groups: age, gender, and the deworming and rearing practices (Table-2). GI parasites were found in almost all study areas. The overall prevalence of feline GI parasites within the Yangon region was 79.56% (183/230) and 57.82% (133/230) was infected with more than one parasite species in the diagnostic stage. All sampled cats were subclinically infected, as they did not show any clinical symptoms at the time of sample collection. Fecal examination results were described using fecal wet mount, centrifugal sedimentation, and centrifugal flotation. The recovery rates of GI parasites according to coprological tests were 33.04% (76/230), 65.21% (150/230), and 42.60% (98/230) for fecal wet mount, centrifugal sedimentation, and centrifugal flotation, respectively. The number of nematodes was recovered from centrifugal sedimentation (n = 153) and centrifugal flotation (n = 102; Table-3).

**Table-2:** Demographic data of sampled cats.

Characteristics	Collected sample % (n)
Age	
<1 year	28.26 (65/230)
1–3 year	45.22 (104/230)
>3 year	26.52 (61/230)
Gender	
Male	42.60 (98/230)
Female	57.40 (132/230)
Deworming practice	
Yes	26.52 (61/230)
No	73.48 (169/230)
Rearing practice	
Access to outdoor	80 (184/230)
Not access to outdoor	20 (46/230)

**Table-3:** The different recovery rate of gastrointestinal parasites according to different coprological tests.

Diagnostic methods	Positive % (n)
Wet fecal mount	33.04 (76/230)
Ethyl acetate centrifugal sedimentation	65.21 (150/230)
ZnSO4 centrifugal flotation	42.60 (98/230)

**Figure-2:**
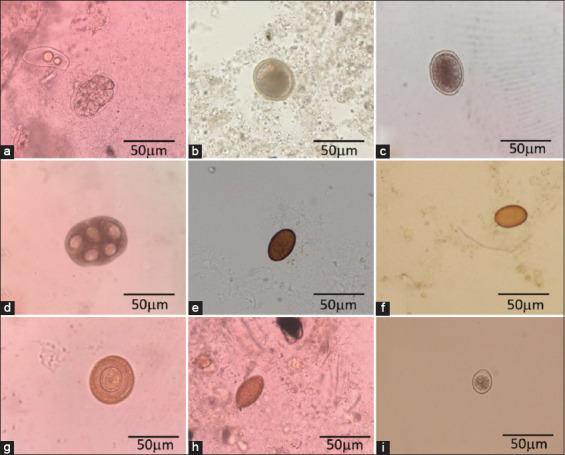
The fecal eggs of gastrointestinal parasites in owned and sheltered cats within Yangon region. (a) *Ancylostoma* spp. Egg, (b and c) *Toxocara* spp. eggs, (d) *Dipylidium caninum* egg, (e and f) *Platynosomum* spp. egg, (g) *Taenia* spp. egg, (h) *Trichuris* spp. egg, (i) *Cystoisospora* spp. egg.

The prevalence rate for parasites, such as cestodes, nematodes, trematodes, and coccidia, was reported (Table-4). Seven parasitic genera were found, including *Ancylostoma* spp., *Toxocara* spp., *Trichuris* spp., *Platynosomum* spp., *Dipylidium caninum*, *Taenia* spp., and *Cystoisospora* spp. (Figure-2). *Ancylostoma* spp. was highest at 55.65% (128/230), but a microscopic examination could not identify the species level of the parasite. About 46.08% (106/230) of sampled cats were infected with *Toxocara* spp. Soil-transmitted helminths of *Trichuris* spp. infected 20.86% (48/230) of sampled cats. Sampled cats were infected with *Platynosomum* spp. [11.73% (27/230)], *D*. *caninum* [7.39% (17/230)], and *Taenia* spp. [4.34% (10/230)]. *Cystoisospora* spp. was also found in 32.17% (74/230) of sampled cats.

**Table-4:** The prevalence rate for different parasites such as cestodes, nematodes, trematodes, and coccidia among owned and sheltered cats from Yangon, Myanmar.

Species infected	Positive % (n)	Diagnosis method
Nematodes		
*Ancylostoma* spp.	55.65 (128/230)	FWM, sedimentation, and flotation
*Toxocara* spp.	46.08 (106/230)	sedimentation and flotation
*Trichuris* spp.	20.86 (48/230)	sedimentation
Cestodes		
*Dipylidium caninum*	7.39 (17/230)	sedimentation
*Taenia* spp.	4.34 (10/230)	sedimentation
Trematodes		
*Platynosomum* spp.	11.73 (27/230)	sedimentation
Protozoa		
*Cystoisospora* spp.	32.17 (74/230)	sedimentation and flotation
Coinfection with two parasites	57.82 (133/230)	FWM, sedimentation, and flotation
Coinfection with three parasites	12.17 (28/230)	FWM, sedimentation, and flotation
Coinfection with more than three parasites	6.52 (15/230)	FWM, sedimentation, and flotation

FWM=Fecal wet mount, Sedimentation: Ethyl acetate centrifugal sedimentation, Flotation: ZnSO_4_ centrifugal flotation

Regarding associated factors, age and gender were not associated with GI parasites. However, domestic cats between 1 and 3 years were more affected by GI parasites, but the difference was not significant (p > 0.05). As a result, the deworming practice was significantly associated (p < 0.05) with GI parasitic infections in owned and sheltered cats. Shelter cats showed a higher parasite burden than owned cats. In this study, a cat with the previous anthelmintic treatment within 6 months had less chance of parasitic infestation. In addition, rearing practice was significantly associated (p < 0.05) with infection. Shelter and owned cats with ready access to outdoors showed a higher prevalence than confined ones. Cats in hall-type shelters and owned cats with easy access to outdoors could easily get exposed to fecal parasite eggs and infected by others and vice versa (Table-5).

**Table-5:** Analysis of risk factors associated with GI parasite infection amongst domestic owned and sheltered cats from Yangon, Myanmar.

No.	Risk factors	Positive to GI parasites % (n)	p-value (95%CI)
1.	Gender		0.061 (1.98–3.22)
	Male	79.59 (78/98)	
	Female	79.54 (105/132)	
2.	Age		0.07 (1.04–3.7)
	<1 year	70.76 (46/65)	0.19 (1.02–4.1)
	1–3 year	95.19 (99/104)	
	>3 year	62.29 (38/61)	
3.	Deworming practice		0.05* (11.12–23.63)
	Yes	96.72 (59/61)	
	No	73.37 (124/169)	
4.	Rearing practice		0.03* (31.03–54.61)
	Access to outdoor	88.04 (162/184)	
	Not access to outdoor	45.65 (21/46)	

* Had association with the presence of GI parasites. GI=Gastrointestinal, CI=Confidence interval

## Discussion

GI parasitism in cats is a major worldwide health problem [23]. This study aimed to determine GI parasitism in cats within the Yangon region, the most populated area for rearing companion animals. For this purpose, three coprological tests, including fecal wet mount, ethyl acetate centrifugal sedimentation, and zinc sulfate centrifugal flotation, were used. In addition, significant factors with GI parasites were revealed. Kittens <6 months were not included in this study due to their immature immune system [24]. The overall prevalence of GI parasites was 79.56% (183/230), in agreement with Ngui *et al*. [13], with 89.3% of GI parasites detected in Malaysia.

In contrast, this finding showed a remarkably high prevalence compared to others: 68.33% in Indonesia [15], and 44.3% in Thailand [17]; the climatic condition was probably similar to the current study area. As mentioned previously, seasonal changes and the host’s immune status favor parasite development and the viability of parasite eggs [25]. Therefore, environmental characteristics could be a predisposing factor for the occurrence of GI parasites in this study. Because people living in the study area share the same environmental characteristics, the importance of zoonosis potential reservoirs should be considered. In this study, even owned cats can be infected if they can freely access the outdoors.

*Ancylostoma* spp. was the most commonly detected parasite in this study, followed by *Toxocara* spp., *Cystoisospora* spp., *Platynosomum* spp., *D. caninum*, and *Taenia* spp. In line with previous studies, these parasites are the most common feline endoparasites [10, 14, 16]. In *Ancylostoma* spp., hookworm infection was the most predominant at 25.0%–78.0% in Malaysia [13], Indonesia [26], and Brazil [27]. It was assumed that the stray cats, before being adopted, might have the diagnostic stage of parasites or become infected by their hunting and grooming practices [10, 28]. To determine GI parasitism in cats, a routine fecal examination should be performed [29].

In this study, cats aged between 1 and 3 years were more infected than others, but there was no significant association. Gates and Nolan [30] revealed that young ones are more susceptible than adult animals. In contrast, a high prevalence rate was shown in older animals [31]. It could be assumed that regular exposure to infected animals might be a possible route to getting an infection. Regarding gender, a high positive number was seen in females with more frequent hunting habits than males [32], but the difference was not significant. The result was in accordance with Lorenzini *et al*. [33], in which gender was insignificant in GI parasites [10].

Depending on significant risk factors, the deworming practice was associated with GI parasites in this study. There was limited knowledge about regular the deworming practices in the study area. Deworming practice is an important preventive care regimen to reduce endoparasites and ectoparasites, preventing zoonotic transmission [15]. In this study, rearing practice had a significant association with GI parasites. In the Yangon area, a year-round moist and warm environment could favor the development of GI parasites, where most sampled cats can also freely access the outdoors to hunt [10].

*Ancylostoma* spp. was the most predominant GI parasite in this study. Similar results have been reported regarding the highest prevalence of hookworm infections among the sampled cats [34–36]. It is difficult to determine the species level of the parasite by microscopic examination; therefore, further molecular studies should be implemented with zoonotic significance. Previously, *Toxocara* spp. was the second most prevalent GI parasite in domestic cats [13]. The fecal eggs of *Toxocara* spp. disperse to the environment and mature, and people get infected, resulting in two clinical syndromes (visceral and ocular larva migrans) [6]. Therefore, it has public health significance due to the high level of prevalence in this study.

*Platynosomum* spp. is the causal organism for lizard poisoning. This parasite resides in the biliary system and gallbladder. A heavy parasite load could lead to the impairment of the biliary system and cholangiocarcinoma. As cats are predators, the prevalence of *Platynosomum* spp. in this study might be due to their hunting behavior. Cats were probably infected by hunting rodents that carry infective stage metacercaria [37]. Among *Trichuris* spp., the two whipworm species, *Trichuris* *serrata* and *Trichuris campanula*, can infect cats [38]. Residual eggs in the environment could be a potential source of infection in this study. With concerns regarding the zoonotic potential of *Trichuris* spp., further molecular studies should be assessed. The prevalence of *Platynosomum* spp. and *Trichuris* spp. could be underestimated in this study, as these parasites are intermittent shedders [38], and fecal sample examination was done from a single sample of each cat.

Based on a previous report, *D. caninum* is the most common tapeworm in companion animals [39]. In this study, cestode eggs of *Taenia* spp. were also detected. Sheltered cats are more likely to get infected due to less access to veterinary care. In this study, owned and sheltered cats were infected with tapeworms due to their grooming habit, and flea intake could be higher, leading to an increased risk of tapeworm infections [40]. The sampled cats in this study were infected with *Cystoisospora* spp. Further evaluation of *Cystoisospora* spp. should be done due to differences in pathogenicity within different species and their zoonotic significance. The previous report of *Cystoisospora* spp. in owned and sheltered cats has been documented [41].

This study documented a remarkably high prevalence of GI parasites in cats within the Yangon region. These results suggest the probability of GI parasites not only due to exposure but also the transmission of other pathogens. Although the prevalence rate was not represented for all regions of Myanmar, this information might help implement possible control measures. Further studies in different regions of Myanmar are needed to determine the overall prevalence of feline GI parasites for a better understanding of parasite epidemiology.

## Conclusion

This study revealed that parasitic infections are common in owned and sheltered cats within the Yangon region. Hookworms and roundworms were the most predominant GI parasites, among others. In addition, significant risks of GI parasitism in cats without deworming under outdoor accessible conditions were determined. There is an urgent need for regular deworming practice, and routine fecal exams with appropriate techniques should be encouraged for zoonotic hookworms and roundworms. Further studies should be performed to evaluate the public health significance of GI parasitic infections in cats.

## Authors’ Contributions

BKS: Conceptualization and methodology. BKS, KSH, and ZHT: Collection of the samples, laboratory work, and drafted the manuscript. TWN: Data analysis. BKS and WM: Supervision of the laboratory work. BKS, TWN, and WM: Manuscript revision. All authors have read, reviewed, and approved the final manuscript.
